# Dopamine D_3_ receptor and GSK3β signaling mediate deficits in novel object recognition memory within dopamine transporter knockdown mice

**DOI:** 10.1186/s12929-019-0613-y

**Published:** 2020-01-03

**Authors:** Pi-Kai Chang, Jung Chu, Ya-Ting Tsai, Yan-Heng Lai, Jin-Chung Chen

**Affiliations:** 1 Department of Physiology and Pharmacology, Graduate Institute of Biomedical Sciences, School of Medicine, Chang Gung University, Taoyuan, Taiwan; 2 Department of Biomedical Sciences, School of Medicine, Chang Gung University, Taoyuan, Taiwan; 3 Department of Medical Imaging and Radiological Sciences, School of Medicine, Chang Gung University, Taoyuan, Taiwan; 4 Healthy Ageing Research Center, Chang Gung University, Taoyuan, Taiwan; 5 Neuroscience Research Center, Chang Gung Memorial Hospital, Linko, Taiwan

**Keywords:** Novel object recognition (NOR), Dopamine, Dopamine D_3_ receptor, Glycogen synthase kinase 3 (GSK3), Medial prefrontal cortex (mPFC), Dopamine transporter knockdown (DAT-KD)

## Abstract

**Background:**

Over-stimulation of dopamine signaling is thought to underlie the pathophysiology of a list of mental disorders, such as psychosis, mania and attention-deficit/hyperactivity disorder. These disorders are frequently associated with cognitive deficits in attention or learning and memory, suggesting that persistent activation of dopamine signaling may change neural plasticity to induce cognitive or emotional malfunction.

**Methods:**

Dopamine transporter knockdown (DAT-KD) mice were used to mimic a hyper-dopamine state. Novel object recognition (NOR) task was performed to assess the recognition memory. To test the role of dopamine D_3_ receptor (D_3_R) on NOR, DAT-KD mice were treated with either a D_3_R antagonist, FAUC365 or by deletion of D_3_R. Total or phospho-GSK3 and –ERK1/2 signals in various brain regions were measured by Western blot analyses. To examine the impact of GSK3 signal on NOR, wild-type mice were systemically treated with GSK3 inhibitor SB216763 or, micro-injected with lentiviral shRNA of GSK3β or GSK3α in the medial prefrontal cortex (mPFC).

**Results:**

We confirmed our previous findings that DAT-KD mice displayed a deficit in NOR memory, which could be prevented by deletion of D_3_R or exposure to FAUC365. In WT mice, p-GSK3α and p-GSK3β were significantly decreased in the mPFC after exposure to novel objects; however, the DAT-KD mice exhibited no such change in mPFC p-GSK3α/β levels. DAT-KD mice treated with FAUC365 or with D_3_R deletion exhibited restored novelty-induced GSK3 dephosphorylation in the mPFC. Moreover, inhibition of GSK3 in WT mice diminished NOR performance and impaired recognition memory. Lentiviral shRNA knockdown of GSK3β, but not GSK3α, in the mPFC of WT mice also impaired NOR.

**Conclusion:**

These findings suggest that D_3_R acts via GSK3β signaling in the mPFC to play a functional role in NOR memory. In addition, treatment with D_3_R antagonists may be a reasonable approach for ameliorating cognitive impairments or episodic memory deficits in bipolar disorder patients.

## Introduction

Bipolar disorder (BD) is often accompanied by progressive impairment in many cognitive components, such as attention, memory and executive function [1], with BD patients showing poor performance on learning and memory tasks especially during the manic phase [2, 3]. The pathology of mania-dependent recognition or episodic memory deficits is known to involve hyper-dopamine (DA) activity and over-stimulation of post-synaptic DA receptors [4, 5]. Due to DA transporter (DAT) titrates the concentration of DA in the synaptic cleft via its uptake activity, reduced DAT function may lead to excessive synaptic DA and the subsequent appearance of bipolar symptoms [6, 7]. DAT-knockdown (KD) mice show a 90% decrease in DAT expression and a 70% increase in extracellular DA concentration in the striatum [8]. As a result, DAT-KD mice exhibit aberrant cortical glutamatergic afferents and display mania-like behaviors [9–11]. Both DAT-KD and wild-type (WT) mice treated with the DA reuptake inhibitor, GBR12909, exhibit deficits in novel object recognition (NOR) memory [12]. Excessive DA tone disrupts attention or object learning, but does not affect consolidation or retrieval of NOR memory. Notably, the deficit in NOR can be prevented by DA D_3_ receptor (D_3_R) deletion or pretreatment with the D_3_R antagonist, FAUC365, suggesting that this receptor may play an essential role in DAT hypo-function-induced NOR impairment [12].

Glycogen synthase kinase 3 (GSK3) contains two different subtypes, GSK3α and GSK3β, encoded by two distinct genes [13]. GSK3 activity is inhibited by various protein kinases that induce a rapid but reversible phosphorylation of GSK3 at S21 or S9 [14], a signaling event known to be downstream of numerous well-known signaling pathways, including insulin, Wnt and Hedgehog [15]. Dysregulation of GSK3 activity is associated with cognitive impairment in chronic brain disorders, such as BD, schizophrenia, depression, Alzheimer’s disease and Fragile-X syndrome [16]. As such, it has been suggested that GSK3 would be developed as a therapeutic target for these disorders [16]. Overexpression of GSK3β in mice cortical and hippocampal neurons impairs spatial learning and memory performance in the Morris water maze and non-spatial memory in the NOR task [17, 18]. Moreover, shRNA knockdown of GSK-3β in the dentate gyrus results in a deficit of contextual fear memory [19], suggesting a dynamic function of GSK3 in distinct learning and memory processes.

The current study aims to further elucidate the molecular events that contribute to DAT-KD-induced deficits in the NOR test. We first examined Akt/GSK3 and ERK1/2 signals in various brain regions of WT and DAT-KD mice exposed to novel objects. Phosphorylation of GSK3 in the medial prefrontal cortex (mPFC) was decreased after novelty exposure in WT but not DAT-KD mice. Furthermore, DAT-KD mice treated with FAUC365 recovered novelty induced-dephosphorylation of GSK3, and a similar result was found with D_3_R-deficient mice. Finally, we observed poor NOR performance in WT mice treated with a systemic GSK3 inhibitor, SB216763, or after administration of GSK3β shRNA to the mPFC.

## Methods

### Animals

Male DAT-KD and age-matched WT C57BL/6 (B6) mice used in this study were tested approximately 10–12 weeks of age. D_3_R-KO/DAT-KD double mutant mice were bred from heterozygote-heterozygote crosses of D_3_R-KO (B6.129S4-Drd3^tm1Dac^/J) and DAT-KD mice [12]. DAT-KD and D_3_R-KO were backcross-bred with C57BL/6 more than 10 generations. WT B6 mice were purchased from the National Laboratory Animal Center, Taiwan. All experimental mice were group-housed under standard conditions, i.e. 70% humidity, 25^∘^C and 12:12 light/dark cycle (light on at 7:00 and off at 19:00) in the Chang-Gung Animal Core Facility. Animal experiments were all performed at the light cycle. All the experimental procedures were conducted in accordance with the guidelines of Animal Care and Use Committee at Chang-Gung University (CGU11–123).

### Novel object recognition (NOR) task

The NOR task was carried out according to our previous study [12]. Briefly, all mice habituated in NOR arena for 5 min in the absence of testing objects for three consecutive days. On the fourth day, the mice were explored two identical objects at different corners of the arena for 10 min (training trial). On the fifth day, the mice were then explored a novel object and a familiar object from training trial for 10 min (testing trial). The time that mice spent on exploring familiar and novel objects was manually recorded. The discrimination index (DI) [(time spent exploring the novel object – time spent exploring the familiar object)/time spent exploring both objects] was calculated. Horizontal locomotion and total exploration time were analyzed using an EthoVision video-tracking system (Noldus, Wageningen, The Netherlands). To examine the role of D_3_R in DA hyperactivity-induced NOR deficits, FAUC365 (3 mg/kg, dissolved in dH_2_O with 5% DMSO and 0.3% Tween 80) or vehicle was subcutaneously (s.c.) injected into DAT-KD mice 10 min before the NOR task training trial. To examine the role of GSK3 in the NOR task in WT mice, SB216763 (1–10 mg/kg, dissolved in dH_2_O with 5% DMSO and 0.3% Tween 80) or vehicle was intraperitoneally (i.p.) injected into B6 mice 10 min prior to the NOR task training trial. Finally, to test the involvement of prefrontal GSK3α and GSK3β in the NOR task, the WT mice received lentivirus injections 7 days before starting habituation for the NOR task.

### Western immunoblots

Mice were placed in an open field arena without the test objects (control group) or with two identical objects (exposed group) for 10 min. Afterwards, mice were decapitated immediately, and brain regions were rapidly dissected and frozen in liquid nitrogen. For the GSK3α/β-KD experiment, the mice were sacrificed after the NOR task (12th day after lentiviral injection), with designated brain regions rapidly dissected and frozen in liquid nitrogen. Brain tissues were homogenized in an ice-cold 1% SDS solution. The protein concentrations were quantified by the Bradford protein assay. Protein extracts were mixed with sample buffer and denatured at 100 ^∘^C for 5 min. The samples containing 20 μg protein were separated by SDS-PAGE (10% acrylamide) and transferred onto hydrphobic polyvinylidene difluoride membranes. Membranes were blocked for 1 h in the Tris-buffered saline plus 0.1% Tween-20 (TBS-T) containing 5% nonfat milk, and incubated overnight at 4^∘^C with primary antibody diluted in TBS-T. Designated protein species were detected using a specific primary antibody: phospho-ERK1/2 (1:1000 dilution, #9101, Cell Signaling, Danvers, MA, USA), total ERK (1:1000 dilution, #9102, Cell Signaling), phospho-Ser9 GSK3β (1:1000 dilution, #5558, Cell Signaling), phospho-Ser9/21-GSK3α/β (1:1000 dilution, #9327, Cell Signaling), total GSK3α/β (1:1000 dilution, sc-7291, Santa Cruz Biotechnology Inc., Dallas, TX, USA), phospho-Ser473-Akt (1:2000 dilution, #4060, Cell Signaling), total Akt (1:1000 dilution, #9272, Cell Signaling) or GAPDH (1:5000 dilution, GTX100118, GeneTex, Irvine, CA, USA), followed by horseradish peroxidase conjugated goat anti-rabbit secondary antibody (1:2000 dilution, GE Healthcare, Chicago, IL, USA). Protein immunoreactive signals were detected by a ChemiDoc™ XRS+ System (Bio-rad, Hercules, CA, USA) and quantified via the Image Lab™ system (Bio-rad).

### Stereotaxic injections of lentivirus constructs

Viral titers of LacZ: 1.7 × 10^7^ RIU/ml, GSK3α: 3 × 10^7^ RIU/ml and GSK3β: 2.5 × 10^7^ RIU/ml (RNAi core, Academia Sinica, Taiwan), were used for in vivo stereotaxic injections. Lentivirus was thawed on ice and resuspended by pipetting before use. A mixture of 80 mg/kg ketamine and 40 mg/kg xylazine solution was intraperitoneally injected to anesthetize the animals. Lentivirus was stereotaxically injected into the mPFC using a 30-gauge blunt-tip needle connected to a syringe (Hamilton Robotics, Reno, NV, USA) by a poly-ethylene catheter. The stereotaxic coordinate for the mPFC was AP: + 1.9 mm, ML: 0.5 mm, DV: − 0.28 mm relative to the bregma. Each mouse was injected bilaterally (1 μl each side), receiving vectors encoding shRNA directed against GSK3α, GSK3β or LacZ. Meloxicam (Sigma Aldrich, St. Louise, MO, USA) was administered (5 mg/kg, s.c.) for 2 days after surgery to relieve pain from the surgical operation. The NOR task was carried out 7 days after surgery.

### Data analysis

All data are expressed as the mean ± SEM. Exploration data in the NOR task were analyzed by a two-way ANOVA followed by Sidak’s post hoc comparisons when significance was identified by the ANOVA. The DI in the NOR task and western blot data were analyzed using a Student’s t-test or a one-way ANOVA followed by the Dunnett’s or Tukey’s post hoc comparisons, when appropriate. All statistics were performed by GraphPad Prism™ (San Diego, CA, USA).

## Results

### D_3_R blockade or deletion reverses DAT-KD-induced NOR deficit

**Fig. 1:**
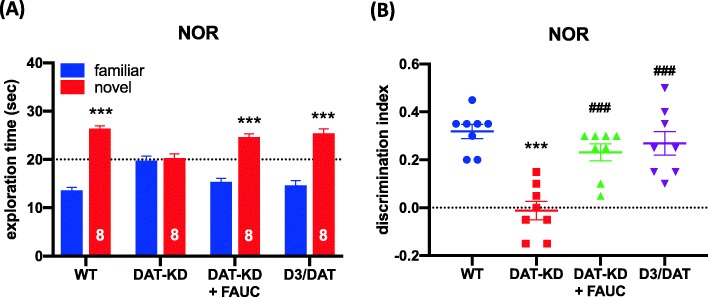
Effects of D_3_R blockade or deletion on DAT-KD-induced NOR deficit. **a** Time that DAT-KD, FAUC365-treated DAT-KD, D_3_R-KO/DAT-KD mutant and WT mice spent on exploring a novel and a familiar objects in the testing trial of the NOR test. *** *p* < 0.001. **b** Discrimination index (DI) for DAT-KD, FAUC365-treated DAT-KD, D_3_R-KO/DAT-KD mutant and WT mice. *** *p* < 0.001 compared to the WT group; ### *p* < 0.001 compared to the DAT-KD group (*n* = 8 per group)

To validate our previous finding that DAT-KD induces D_3_R-dependent object recognition deficits, a NOR test was administered to WT, DAT-KD, FAUC365-treated DAT-KD (3 mg/kg, s.c., 10 min before training) and D_3_R-KO/DAT-KD double mutant mice. Two-way ANOVA of the NOR test exploration data for different experimental groups of mice revealed significant main effects of the object (F_1,56_ = 204.8, *p* < 0.001) and a significant object × group interaction (F_3,56_ = 21.63, *p* < 0.001). Sidak’s post hoc comparisons showed that the WT, FAUC365-treated DAT-KD and D_3_R-KO/DAT-KD mutant mice spent more time exploring novel objects than familiar objects (*p* < 0.001), but the DAT-KD mice did not (Fig. 1a). A one-way ANOVA showed that the group effect on DI was significant (F_3,28_ = 14.36, *p* < 0.001; Fig. 1b). In the *post-hoc* analysis, the DI of DAT-KD mice was lower than that of WT mice. The DI in the FAUC365 (3 mg/kg)-treated DAT-KD and the D_3_R-KO/DAT-KD mutant mice was higher than that of DAT-KD mice (*p* < 0.001). There were no differences in time spent exploring the two identical objects or total object exploration during the training trial (*p* > 0.05; Additional file 1: Figure S1A & B). The DAT-KD mice showed a higher horizontal locomotor activity compared to the WT mice during the NOR training trial (*p* < 0.05), but locomotion was not affected by D_3_R blockade or deletion (*p* > 0.05; Additional file 1: Figure S1C).

### Effects of DAT-KD on Akt/GSK3 and ERK1/2 signaling in various brain regions after exposure to novelty

**Fig. 2:**
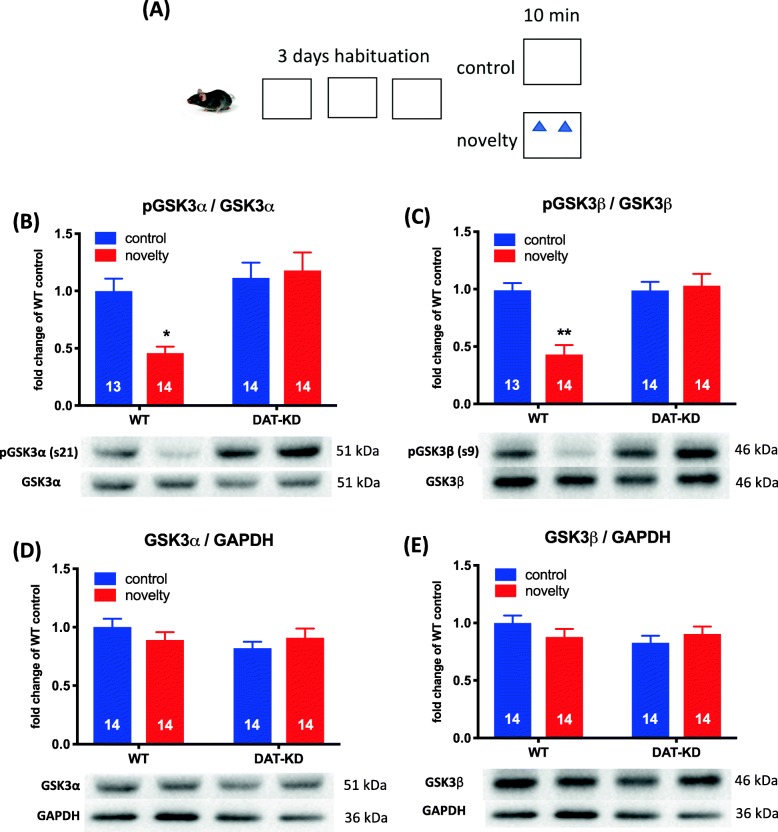
DAT-KD mice do not exhibit diminished GSK3α/β phosphorylation in the mPFC after novelty exposure. **a** Schematic representation of the experiments to evaluate DA signaling effects after novel object exposure. After 3 days of habituation, mice were allowed to explore two equal novel objects for 10 min, followed immediately by euthanization. **b** Levels of phosphorylation at serine 21 of GSK3α; **c** Levels of phosphorylation at serine 9 of GSK3β; **d** total amount of GSK3α and **e** GSK3β. Data are shown as mean ± SEM (*n* = 13–14 per group). *p < 0.05, **p < 0.01 compared to the WT control group

We next sought to identify the CNS location of DA signaling pathways involved in NOR-related cognition by analyzing tissues from discrete brain regions with western blot. DAT-KD and WT mice were placed in a NOR arena with objects (exposed group) or without objects (control group) for 10 min (Fig. 2a); then, mice were euthanized for analysis. Since Akt/GSK3 and ERK1/2 signals account as most notable signal transduction pathways in association with Go/Gi-coupled D_3_R [20], Akt/GSK3 and ERK1/2 signals in the mPFC, dorsal hippocampus (DH) and ventral striatum (VS) were analyzed. In the mPFC, two-way ANOVA showed a significant main effect of novelty exposure (F_1,51_ = 11.73, *p* < 0.001) and a significant novelty exposure × genotype interaction (F_1,51_ = 6.235, *p* < 0.01) in the amount of phosphorylated GSK3α (Fig. 2b). The post hoc analysis indicated that WT mice exhibited decreased GSK3α phosphorylation in the mPFC after novelty exposure, while no difference was observed in the DAT-KD mice (Fig. 2b). Similar statistical outcomes were found when analyzing the phosphorylation level of GSK3β, i.e., significance in novelty exposure (F_1,51_ = 9.519, *p* < 0.01); genotype (F_1,51_ = 12.74, *p* < 0.001); and a novelty exposure × genotype interaction (F_1,51_ = 12.86, *p* < 0.001) (Fig. 2c). The post hoc analysis showed a decreased level of GSK3β phosphorylation in the WT mice after novelty exposure (Fig. 2c). No change was observed in the total amounts of GSK3α and GSK3β among the four testing groups (*p* > 0.05, Fig. 2d & e). There were also no apparent differences in the amount of phosphorylated Akt and ERK or the corresponding total proteins in the mPFC (*p* > 0.05, Additional file 2: Figure S2). Moreover, the amount of phosphorylated Akt/GSK3 and ERK1/2 and the corresponding total proteins were not different in the DH and VS (Additional file 3: Figure S3, Additional file 4: Figure S4, Additional file 5: Figure S5, Additional file 6: Figure S6).

### D_3_R deletion and antagonism restore diminished phosphorylation of GSK3α/β in the mPFC of DAT-KD mice

**Fig. 3:**
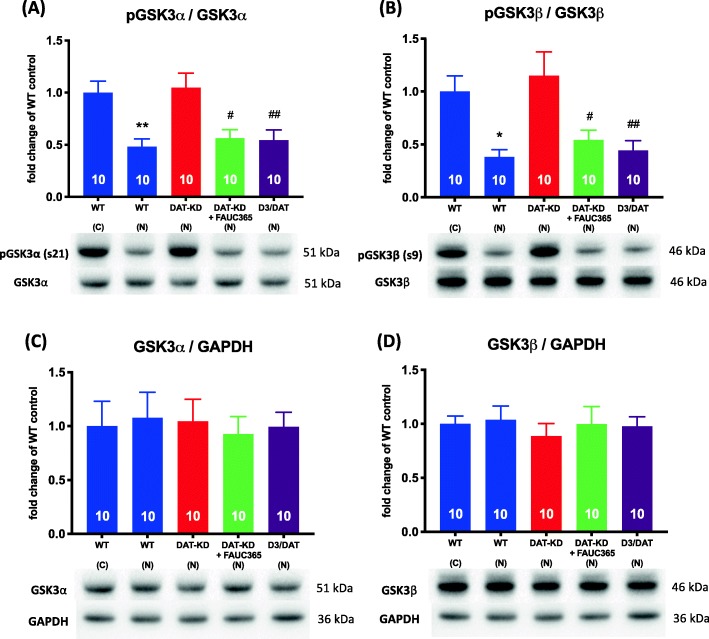
D_3_R inhibition or deletion rescues GSK3 phosphorylation decrease in mPFC after novelty exposure. **a** Levels of phosphorylation at GSK3α/serine 21; **b** Levels of phosphorylation at GSK3β/serine 9; **c** total amount of GSK3α; **d** total amount of GSK3β. Data are shown as mean ± SEM (*n* = 10 per group). *p < 0.05, **p < 0.01 compared to the WT control (c) group; #*p* < 0.05, ##*p* < 0.01 compared to the DAT-KD mice novelty (n) group (*n* = 10 per group)

According to our previous work, the DAT-KD-induced deficit in NOR can be rescued by D_3_R deletion or antagonism [12]. In order to examine whether this rescued deficit is mediated through GSK3 signaling in the mPFC, we measured GSK3α and GSK3β phosphorylation among WT, DAT-KD, FAUC365-treated DAT-KD and D_3_R-KO/DAT-KD double mutant mice after exposure to novelty. One-way ANOVA revealed a significant treatment effect (F_4,45_ = 7.077, *p* < 0.001, Fig. 3a) in the level of GSK3α phosphorylation, and Tukey’s post hoc analysis confirmed that GSK3α phosphorylation was decreased in the WT mice after novelty exposure, but not in the DAT-KD mice. Furthermore, phosphorylation levels of GSK3α in the FAUC365-treated DAT-KD mice (*p* < 0.05) and the D_3_R-KO/DAT-KD double mutant mice were lower than that in the DAT-KD mice (*p* < 0.01; Fig. 3a). Similar results were found with regard to GSK3β phosphorylation, where one-way ANOVA showed a treatment effect (F_4,45_ = 6.403, *p* < 0.001; Fig. 3b). Post hoc analysis showed lower levels of GSK3β phosphorylation in the D_3_R-KO/DAT-KD double mutant (*p* < 0.01) and FAUC365-treated DAT-KD mice compared to the DAT-KD mice (*p* < 0.05; Fig. 3b). There were no differences in the phosphorylation levels of Akt or the total amounts of Akt (Additional file 7: Figure S7), GSK3α or GSK3β among experimental groups (*p* < 0.05; Fig. 3c & d).

### Systemic GSK3α/β inhibition causes deficits in the NOR task in WT mice

**Fig. 4:**
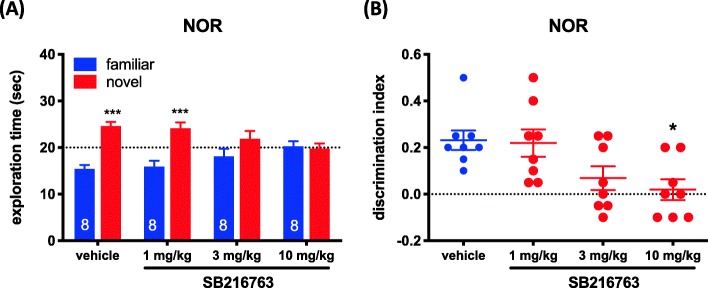
SB216763 induces NOR deficits in WT mice. **a** Effect of SB216763 on exploring a novel and a familiar objects in the retention trial of the NOR task. Data are shown as mean ± SEM (*n* = 8 per group). ***p < 0.001. **b** Effect of SB216763 on DI. Data are shown as mean ± SEM (*n* = 8 per group). *p < 0.05 compared to the vehicle group

Phosphorylation of serine 21 in GSK3α and serine 9 in GSK3β results in inhibition of GSK3α/β activity [21]. C57BL/6 mice were treated with GSK3 inhibitor, SB216763, to determine whether or not GSK3α/β activity is required in the NOR task. In the testing trial, two-way ANOVA showed a significant main effect of object (F_1,58_ = 32.59, *p* < 0.001) and a significant object × treatment interaction (F_3,58_ = 5.916, *p* < 0.01) in the time spent on exploring the novel and familiar objects (Fig. 4a). Sidak’s post hoc comparisons showed no preference for the novel object in the 3 mg/kg and 10 mg/kg groups (*p* < 0.01, Fig. 4a). By ANOVA, SB216763 1, 3 and 10 mg/kg decreased DI significantly (F_3,28_ = 4.693, *p* < 0.01), but by post hoc analysis, DI only decreased at 10 mg/kg (*p* < 0.05, Fig. 4b). No differences in time spent exploring the two identical objects, total exploration time or locomotor activity were observed during the training trial period (Additional file 8: Figure S8).

### Knockdown of GSK3β in the mPFC causes NOR deficits in WT mice

**Fig. 5:**
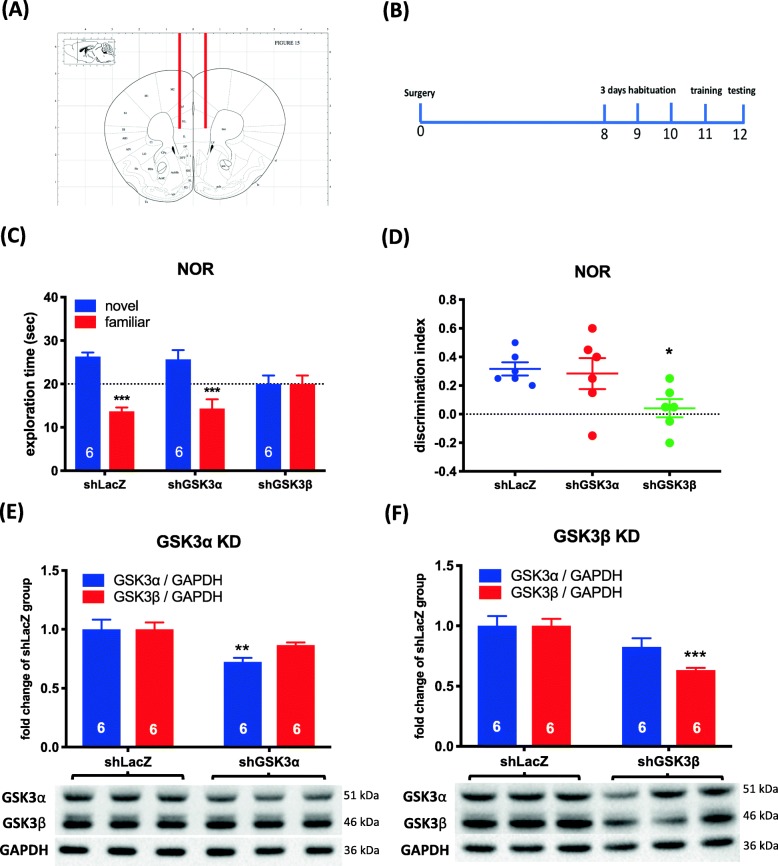
Effects of knocking down GSK3α or GSK3β on the NOR task in mice. **a** Stereotaxic coordinates for lentiviral injections. **b** Timeline of the surgery and behavioral analysis. **c** Effect of knocking down GSK3α or GSK3β on exploring a novel and a familiar objects in the retention trial of the NOR task. Data are shown as mean ± SEM (*n* = 6 per group). ***p < 0.001. **d** Effect of knocking down GSK3α or GSK3β on DI. Data are shown as mean ± SEM (*n* = 6 per group). *p < 0.05, compared with shLacZ group. **e** Knockdown efficiency of shGSK3α and **f** shGSK3β. Data are shown as mean ± SEM (*n* = 6 per group). **p < 0.01, ***p < 0.001 compared to the shLacZ group

To explore the involvement of specific GSK3 isoforms in the mPFC during the NOR task, expression of GSK3α and GSK3β were individually knocked down via microinjection of shRNA-carrying lentivirus to the mPFC of WT mice (Fig. 5a). The NOR task was carried out 7 days after viral injection (Fig. 5b). No difference was observed in the time spent on exploring two identical objects (*p* > 0.05; Additional file 9: Figure S9). Two-way ANOVA showed a main effect of the object (F_1,30_ = 30.57, *p* < 0.001) and an object × treatment interaction (F_2,30_ = 7.712, *p* < 0.01; Fig. 5b). Sidak’s post hoc comparisons showed that the group receiving GSK3β shRNA had no preference for novel objects (*p* > 0.05; Fig. 5c). One-way ANOVA showed that there was a treatment effect on the DI (F_2,15_ = 3.798, *p* < 0.05; Fig. 5d). Dunnett’s post hoc testing revealed that the DI was reduced (*p* < 0.001) in mice treated with shGSK3β, while mice treated with shGSK3α showed similar results to the LacZ control group (Fig. 5d). There were no differences in locomotor activity or total object exploration during the training period (*p* > 0.05; Additional file 9: Figure S9). Twelve days after viral injection, the shRNAs toward GSK3α and GSK3β caused a respective decrease in expression of the two proteins (Fig. 5e and f).

## Discussion

In this study, excessive synaptic DA activity in DAT-KD mice may have been responsible for reduced DI in the NOR task as compared to WT mice. Treatment with the D_3_R antagonist, FAUC365, prevented this impairment in NOR performance, similar to the effect of D_3_R knockout in the D_3_R-KO/DAT-KD double mutant mice. WT mice exhibited reduced phosphorylation of GSK3α (Ser21) and GSK3β (Ser9) in the mPFC after exposure to a novel object, an effect that was not observed in the DAT-KD mice. This loss of novelty-induced GSK3 activity in the mPFC of DAT-KD mice could also be rescued by treatment with FAUC 365 or deletion of D_3_R. Moreover, impaired NOR memory was observed in WT mice treated with SB216763 or knock-down of GSK3β in the mPFC. These findings suggest that D_3_R activation contributes to hyper-DA activity-induced impairment in object learning through the disruption of GSK3β activity in the mPFC.

DAergic neurons originate from the ventral tegmental area that project to the mPFC, VS and hippocampus. This dopaminergic pathway is known to participate in novelty detection and memory processing, as well as attention and motivation [22]. The perirhinal cortex plays a critical role in processing the information of the object identity [23], while the dorsal hippocampus takes charge of not only the spatial learning of the encoded object [24] but episodic memory formation and recollection [25, 26]. Bilateral lesions of the mPFC or contralateral disconnection of the mPFC and perirhinal cortex display intact NOR, but impaired object-place recognition, suggesting that the mPFC is important for the integration of the object with spatial information [27]. However, bilateral inhibition of neural activity in either the dorsal hippocampus or the mPFC alone, or both brain regions via Designer Receptors Exclusively Activated by Designer Drugs (DREADDs) disrupt consolidation of NOR and object-place recognition [28]. This suggests that the mPFC might play a top-down role in affecting NOR via the medial temporal lobe structures (perirhinal, entorhinal and inferior temporal cortices) and the hippocampus in regulating memory processing [24]. Intracellular signaling pathways in the mPFC (or PFC), for example, ERK1/2 and PKMζ, are involved in the acquisition of recognition memory [29, 30]. Furthermore, a novel environment increases DA and noradrenaline release in the PFC and hippocampus [31]. This effect may also occur upon novel object exposure, which in turn affects GSK3 signaling. Phosphorylation of GSK3 in the cerebral cortex increases after 1 week of amphetamine or methylphenidate treatment [32], possibly caused by drug-evoked excessive DA release in the cortex. However, DAT-KD mice did not exhibit increased basal phosphorylation of GSK3; rather, the animals had blunted novelty-induced GSK3 activation/dephosphorylation, suggesting that DA hyperactivity may actually prevent GSK3 activation in the mPFC. After novelty exposure, the FAUC365-treated DAT-KD and D_3_R-KO/DAT-KD double mutant mice both exhibited decreased phosphorylation of GSK3, similar to that seen in WT mice. This clear result strongly suggests that the blunted activation of GSK3 in the mPFC of the DAT-KD mice requires D_3_R activation. It was previously reported that short-term (1–15 min) activation of D_3_R increased Akt and/or GSK3 phosphorylation; as such, a transient increase in phosphorylated GSK3β in mouse striatum followed by systemic administration of quinelorane, a D_2_R/D_3_R agonist, with a maximal effect observed10 min after injection, but this effect was not found in D_3_KO mice [33]. Similar results have been found in Chinese hamster ovary (CHO) cells that overexpress human D_3_R, in which increases in phosphorylation of Akt (Thr308 and Ser473) and GSK3β (Ser9) were observed after short-term incubation of DA (the maximum effect, 5–10 min) [34]. Together, these findings lead us to speculate that transient D_3_R stimulation may increase GSK3β phosphorylation/inactivation to compete with novelty-induced GSK3 activation in the DAT-KD mice. Although D_3_R possesses high DA binding affinity, prefrontal D_3_R is probably not highly occupied by endogenous DA, since FAUC365 does not alter the DI in a D_3_R-selective dose range in WT mice [12].

Our data show that D_3_R plays a modulatory role in NOR. The D_3_R-mediated transient phosphorylation of GSK3β can be substantially activated under hyper-DA conditions (i.e., DAT-KD mice), but not at normal physiological conditions (i.e., WT mice). The current findings thus provide a novel cellular mechanism to explain the previously reported pro-cognitive effect of D_3_R antagonism [35]. Interestingly, the activation of DA D_1_R in the PFC is required for NOR memory encoding, and microinjection of a D_1_R antagonist, SCH23390, before training impairs performance in the NOR task [36]. GSK3β physically interacts with D_1_R in the PFC, and inhibition of GSK3β coincidently reduces the D_1_R-GSK3β association and D_1_R activation [37]. It may therefore be speculated that D_3_R-induced phosphorylation of GSK3β could also disrupt the D_1_R-GSK3β association, leading to an attenuation of D_1_R activation in the mPFC of the DAT-KD mice during training and consequent impairment of NOR task performance. Surprisingly, phosphorylation of ERK1/2 in the PFC was previously reported to be increased after 10 min of novelty exposure [29, 38], but a change in ERK1/2 phosphorylation was not observed in various brain regions in either the WT or the DAT-KD mice in the current study. This discrepancy may be the result of a strain difference (i.e., ICR versus C57BL/6) or brain region difference (i.e., PFC versus mPFC). In addition, we did not observe changes in Akt phosphorylation after novelty exposure in WT mice or DAT-KD mice, suggesting alternative cell signaling events may be responsible for altering GSK3 signaling in the mPFC after NOR task.

Direct activation of GSK3 can occur via dephosphorylation of Ser9/Ser21 by protein phosphatase 1 or, protein phosphatase 2A or 2B, which may be driven by various factors, such as raised calcium level [39]. The role of GSK3 in learning and memory might involve long-term potentiation (LTP) and long-term depression (LTD). Excessive activation of GSK3β inhibits the induction of hippocampal LTP [40, 41], while inhibition of GSK3β blocks the expression of LTD [41], suggesting that the activation of GSK3β plays a key role in regulating synaptic plasticity. Levels of phosphorylated GSK3 in the PFC, nucleus accumbens and hippocampus are reduced 10 min after the re-consolidation of cocaine contextual memory [42]. Moreover, systemic treatment of SB216763 after exposure to a previously cocaine-paired compartment diminishes cocaine-conditioned place preference, implicating that GSK3 suppression disrupts the re-consolidation of cocaine-associated reward memories [42]. In the current study, sustained GSK3β knockdown by shRNA in the mPFC impaired NOR, possibly due to the disruption of retrieval of object memories. Infusion of SB216763 into the mPFC resulted in a reduction in pre-pulse inhibition (PPI) [43]. PPI is a filter that modulates sensory input to the brain, thereby avoiding the sensory overload and cognitive fragmentation [44]. The relationship between PPI and cognition is thought to be mediated by attentional processes. Treatment of FAUC365 prior to training reversed the deficit in NOR in DAT-KD mice. It is possible that D_3_R-mediated GSK3β inhibition similarly affects the processes of sensorimotor gating, attention or motivation, prior to memory encoding.

The therapeutic effects of lithium, the most widely used anti-manic drug for BD, are partly mediated by its action on GSK3 [45]. Treatment of healthy individuals with lithium impairs cognitive performance [46, 47], while treatment of rats with SB216763 (78 pmol/day; icv) for 4 weeks impairs performance in the Morris water maze [48]. Mice treated with SB216763 (2 mg/kg; i.p.) for 2 weeks does not affect performance in contextual memory or spatial learning [49]. The dosage for treating different neurocognitive dysfunctions with elevated GSK3 activity, such as Alzheimer’s disease and fragile X syndrome, may be insufficient to affect basal GSK3 activity in healthy controls. In the current study, the systemic administration of SB216763 (10 mg/kg) before training decreased the DI in the NOR task, suggesting that cognitive performance requires appropriate GSK3 activity.

Manipulating the function of GSK3 isoforms affects NOR performance. Mice with constantly active GSK3β, rather than GSK3α, display a NOR deficit [50]. Studies with transgenic mice indicate that GSK3β is a key regulator of cognitive function. Conditional over-expression of GSK3β in mice cortex and hippocampus impairs performance on the NOR task, and suppression of GSK3β reversed these deficits [18]. Mice with point mutations at GSK3β Ser9 exhibited impaired object recognition memory [51]. Heterozygote GSK3β knockout mice and mice treated with ARA014418, a GSK3 inhibitor, have impaired memory reconsolidation [52]. Moreover, lentiviral knockdown of GSK3β in the dentate gyrus causes abnormal synaptic plasticity and impairment in contextual fear memory [19]. GSK3α deficient mice exhibit impaired NOR suggesting that GSK3α also plays a role in the NOR [53], which is in contrast to our finding, that regional suppression of GSK3α in the mPFC did not sufficiently impair the NOR. In the current study, GSK3β protein levels in the mPFC exhibited a modest but significant reduction after lentiviral injection, but this slight knockdown was sufficient to impair performance on the NOR task. Together, these studies suggest that either low or high GSK3β activity may be harmful to cognitive performance. In addition, GSK3β activity in different brain regions may participate in distinctive cognitive domains.

## Conclusions

The present findings explore an unknown role for GSK3 in the mPFC in the cognitive response to novelty exposure. GSK3β activity in the mPFC seems to be required in executing recognition memory. This finding provides a more complete deciphering the role of GSK3β in learning and memory, and may help to explain why cognitive impairments occur in healthy individuals after administration of GSK3β inhibitors [47]. DA hyperactivity caused by DAT hypo-function disrupted novelty evoked GSK3 dephosphorylation, an effect that could be prevented by D_3_R blockade or knockout. These results provide a mechanistic basis for the rescue effect of D_3_R antagonist on the NOR deficit in DAT-KD mice. Thus, treatment with D_3_R antagonists may be a reasonable approach for ameliorating cognitive impairments or episodic memory deficits in BD patients.

## Supplementary information

**Additional file 1: Figure S1.** Task performance and locomotor activity for DAT-KD, FAUC365-treated DAT-KD, the D_3_R-KO/DAT-KD mutant and WT mice. (A) Time spent on each of two identical objects in 40 s of object exploration during the NOR training trial. (B) Total exploration of objects in the training trial. (C) Cumulative horizontal locomotor activity was recorded for a total of 10 min during the NOR training trial. Data were analyzed by a one-way ANOVA followed by the Tukey’s multiple comparisons test and are shown as mean ± SEM (*n* = 8 per group, * *p* < 0.05 compared to WT mice).

**Additional file 2: Figure S2.** No effect of novelty exposure on Akt and ERK1/2 phosphorylation in the mPFC. (A) Levels of phosphorylation at Akt/serine 473; (B) total amount of Akt. Data are shown as the mean ± SEM (*n* = 13–14 per group). (C) Levels of ERK1 phosphorylation; (D) total amount of ERK1; (E) Levels of ERK2 phosphorylation; (F) total amount of ERK2. Data are shown as mean ± SEM (*n* = 7–8 per group).

**Additional file 3: Figure S3.** No effect of novelty exposure on Akt and GSK3 phosphorylation in the DH. (A) Levels of phosphorylation at Akt/serine 473; (B) total amount of Akt; (C) Levels of phosphorylation at GSK3α/serine 21; (D) total amount of GSK3α; (E) Levels of phosphorylation at GSK3β/serine 9; (F) total amount of GSK3β. Data are shown as mean ± SEM (*n* = 13–14 per group).

**Additional file 4: Figure S4.** No effect of novelty exposure on ERK1/2 phosphorylation in the DH. (A) Levels of ERK1 phosphorylation; (B) total amount of ERK1; (C) Levels of ERK2 phosphorylation; (D) total amount of ERK2. Data are shown as mean ± SEM (*n* = 8 per group).

**Additional file 5: Figure S5.** No effect of novelty exposure on Akt and GSK3 phosphorylation in the VS. (A) Levels of phosphorylation at Akt/serine 473; (B) total amount of Akt; (C) Levels of phosphorylation of GSK3α/serine 21; (D) total amount of GSK3α; (E) Levels of phosphorylation of GSK3β/serine 9; (F) total amount of GSK3β. Data are shown as mean ± SEM (*n* = 13–14 per group).

**Additional file 6: Figure S6.** No effect of novelty exposure on ERK1/2 phosphorylation in the VS. (A) Levels of ERK1 phosphorylation; (B) total amount of ERK1; (C) Levels of ERK2 phosphorylation; (D) total amount of ERK2. Data are shown as mean ± SEM (*n* = 8 per group).

**Additional file 7: Figure S7.** No effect of D_3_R inhibition or deletion on Akt phosphorylation in mice mPFC after novelty exposure. (A) Levels of phosphorylation at Akt/s473; (B) total amount of Akt. (n), novelty exposure; (c) control group (*n* = 10 per group).

**Additional file 8: Figure S8.** Effects of SB216763 on the NOR task in mice. (A) Exploration time up to 40 s during the training trial. (B) Total exploration time spent on two identical objects during the NOR training trial. (C) Cumulative horizontal locomotor activity was recorded for a total of 10 min during the NOR training trial. Data are shown as mean ± SEM (*n* = 8 per group).

**Additional file 9: Figure S9.** Effect of knocking down GSK3α or GSK3β on horizontal locomotion and NOR task in mice. (A) Cumulative horizontal locomotor activity was recorded for a total of 10 min during the NOR training trial. (B) Exploration time up to 40 s during the training trial. (C) Total exploration time spent on two identical objects during the NOR training trial. Data are shown as mean ± SEM (*n* = 6 per group).

**Additional file 10: Figure S10.** Phosphorylation and total GSK3 isoforms in C57BL/6 mice that received 3 mg/kg FAUC365 for 10 min. (A) Phosphorylation at GSK3α/serine 21; (B) Levels of phosphorylation at GSK3β/serine 9; (C) total amount of GSK3α and (D) total amount of GSK3β. Data are shown as the mean ± SEM (*n* = 10 per group).

## Data Availability

On request.

## Abbreviations

BD: Bipolar disorder
CHO: Chinese hamster ovary
CNS: Central nervous system
D_3_R: Dopamine D_3_ receptor
DA: Dopamine
DAT-KD: Dopamine transporter knockdown
DI: Discrimination index
GSK3: Glycogen synthase kinase 3
LTD: Long-term depression
LTP: Long-term potentiation
mPFC: Medial prefrontal cortex
NOR: Novel object recognition
PI-3-K: Phosphatidylinositol-3-kinase
PP1: Protein phosphatase 1
PP2A: Protein phosphatase 2A
PP2B: Protein phosphatase 2B
PPI: Pre-pulse inhibition
TBS-T: Tris-buffered saline plus 0.1% Tween-20
WT: Wild-type
