# Supplementary figures and images for: Dopamine D3 receptor and GSK3β signaling mediate deficits in novel object recognition memory within dopamine transporter knockdown mice
Source: J Biomed Sci. 2020 Jan 3;27:16. doi: 10.1186/s12929-019-0613-y (PMC6942274; doi:10.1186/s12929-019-0613-y)

**(A)**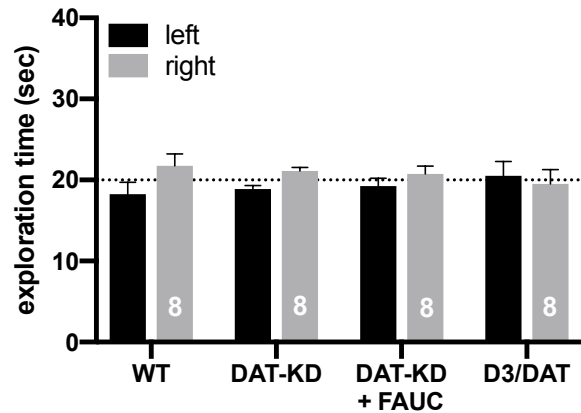**(B)**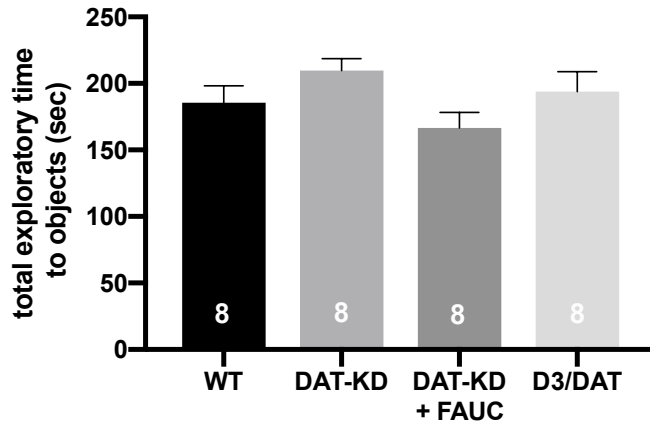**(C)**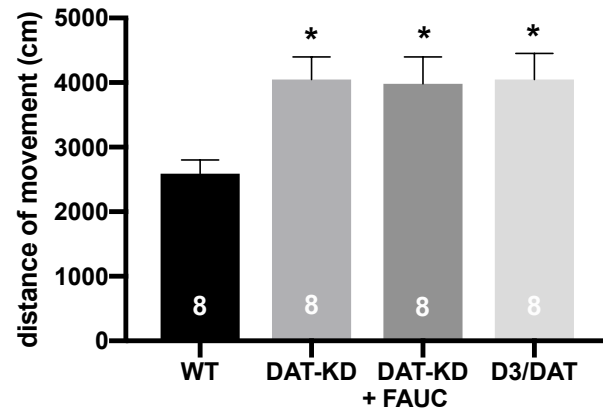

Supplement: Supplementary file 1 — Additional file 1: Figure S1. Task performance and locomotor activity for DAT-KD, FAUC365-treated DAT-KD, the D3R-KO/DAT-KD mutant and WT mice. (A) Time spent on each of two identical objects in 40 s of object exploration during the NOR training trial. (B) Total exploration of objects in the training trial. (C) Cumulative horizontal locomotor activity was recorded for a total of 10 min during the NOR training trial. Data were analyzed by a one-way ANOVA followed by the Tukey’s multiple comparisons test and are shown as mean ± SEM (n = 8 per group, * p < 0.05 compared to WT mice). [file 12929_2019_613_MOESM1_ESM.pdf]

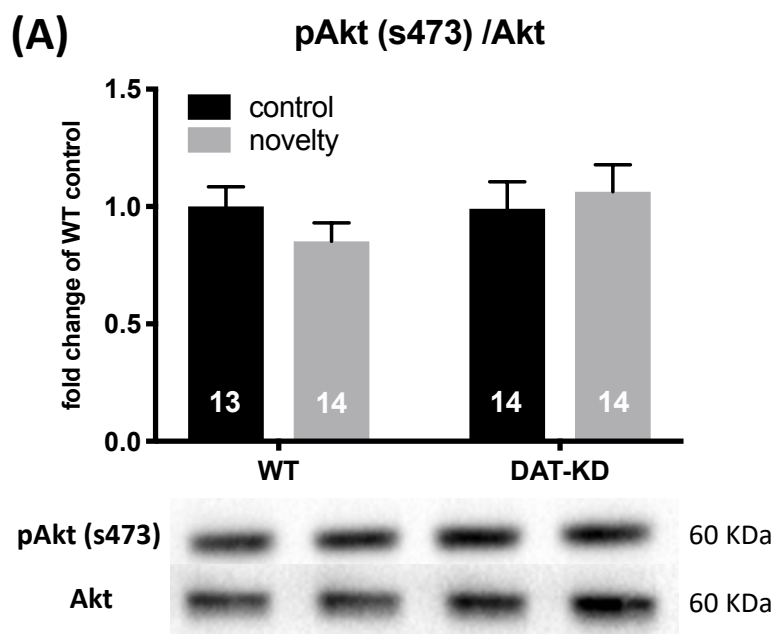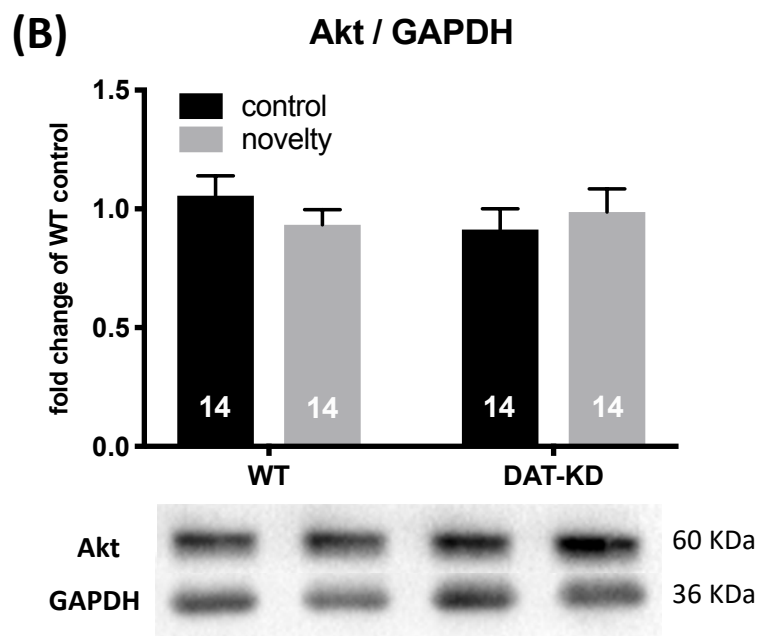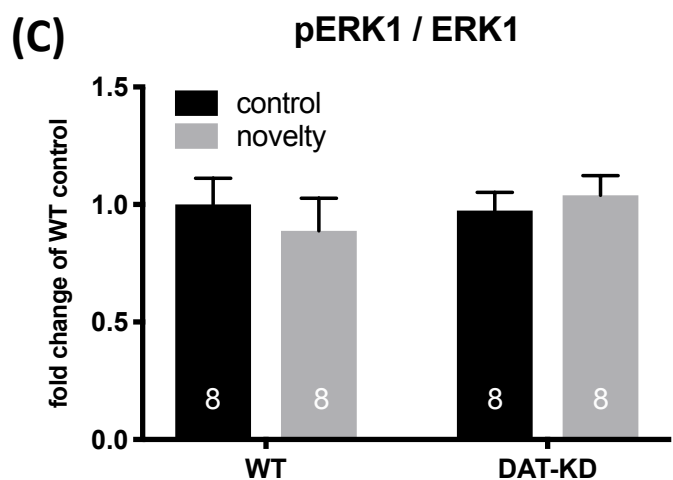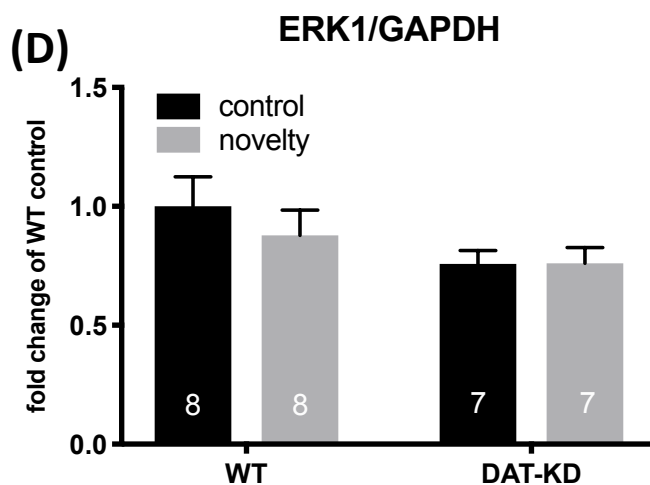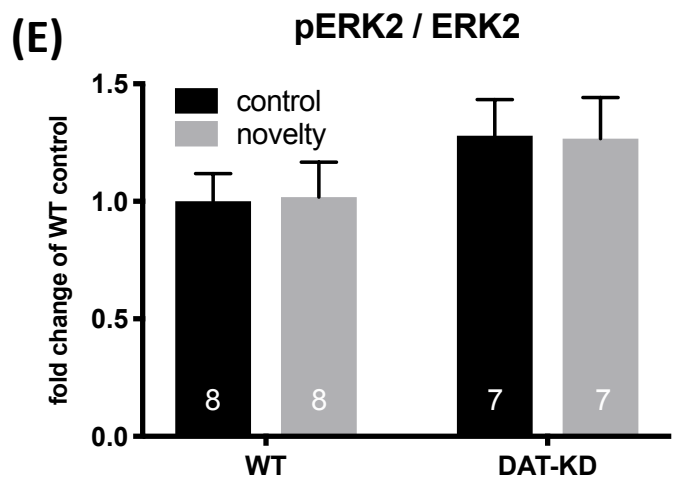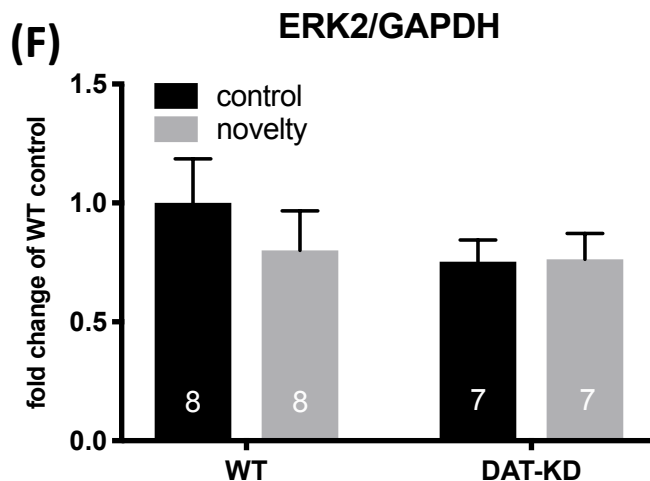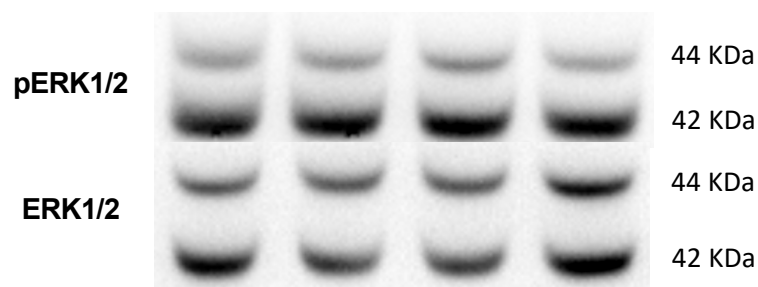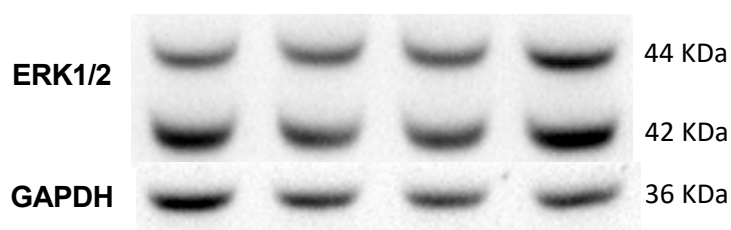

Supplement: Supplementary file 2 — Additional file 2: Figure S2. No effect of novelty exposure on Akt and ERK1/2 phosphorylation in the mPFC. (A) Levels of phosphorylation at Akt/serine 473; (B) total amount of Akt. Data are shown as the mean ± SEM (n = 13–14 per group). (C) Levels of ERK1 phosphorylation; (D) total amount of ERK1; (E) Levels of ERK2 phosphorylation; (F) total amount of ERK2. Data are shown as mean ± SEM (n = 7–8 per group). [file 12929_2019_613_MOESM2_ESM.pdf]

**(A)** pAkt (s473) / Akt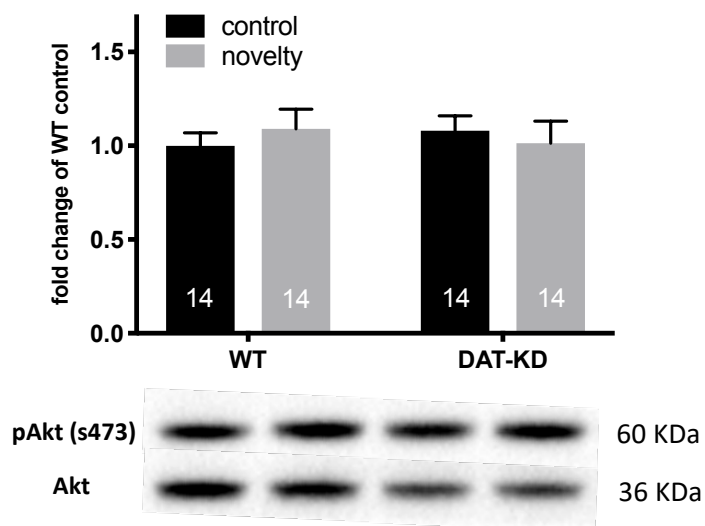**(B)** Akt / GAPDH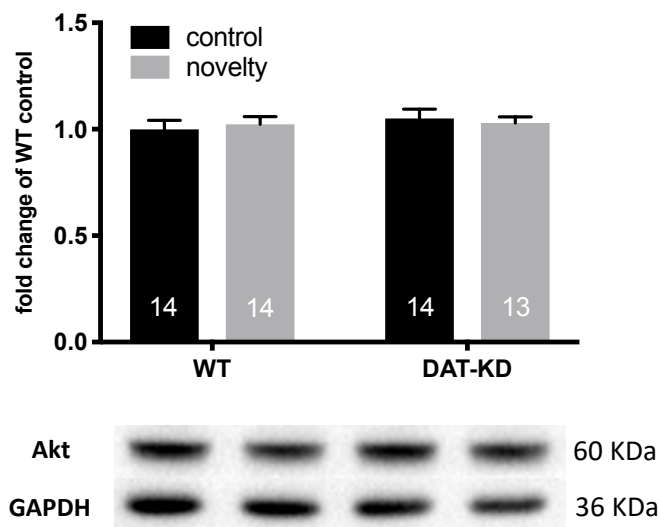**(C)** pGSK3 $\alpha$  / GSK3 $\alpha$ 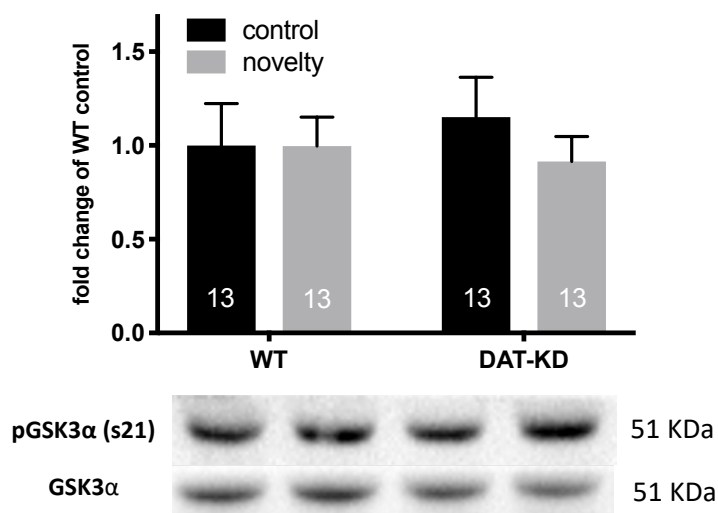**(D)** GSK3 $\alpha$  / GAPDH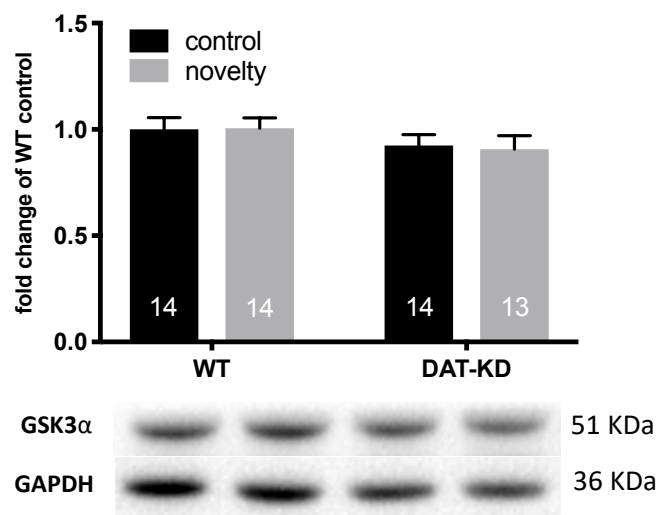**(E)** pGSK3 $\beta$  / GSK3 $\beta$ 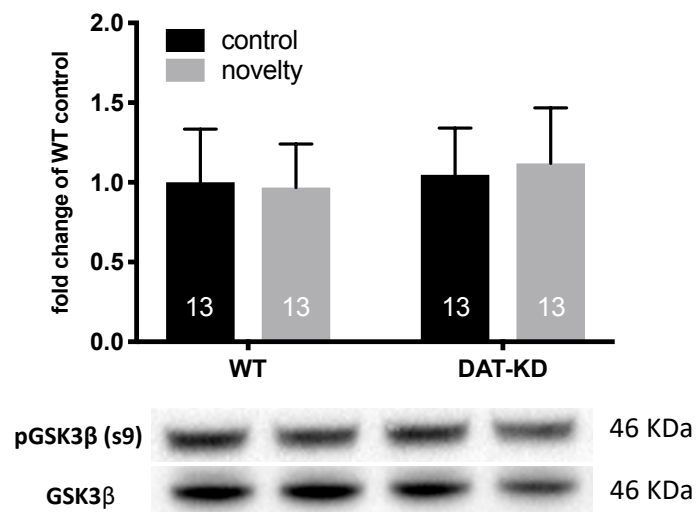**(F)** GSK3 $\beta$  / GAPDH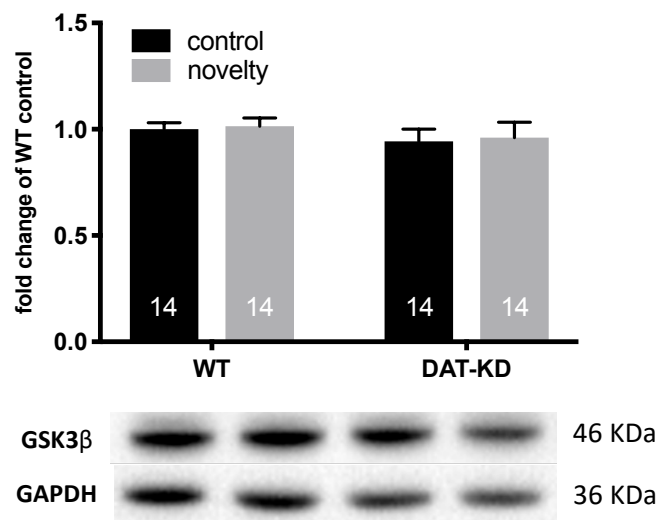

Supplement: Supplementary file 3 — Additional file 3: Figure S3. No effect of novelty exposure on Akt and GSK3 phosphorylation in the DH. (A) Levels of phosphorylation at Akt/serine 473; (B) total amount of Akt; (C) Levels of phosphorylation at GSK3α/serine 21; (D) total amount of GSK3α; (E) Levels of phosphorylation at GSK3β/serine 9; (F) total amount of GSK3β. Data are shown as mean ± SEM (n = 13–14 per group). [file 12929_2019_613_MOESM3_ESM.pdf]

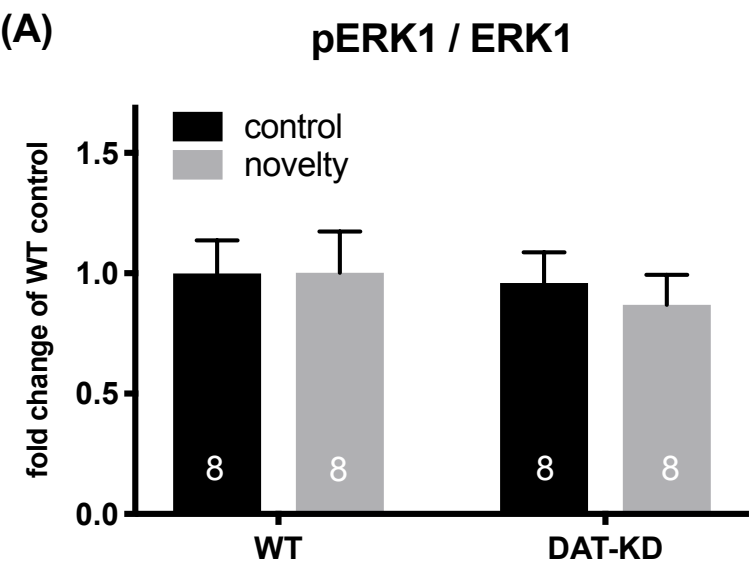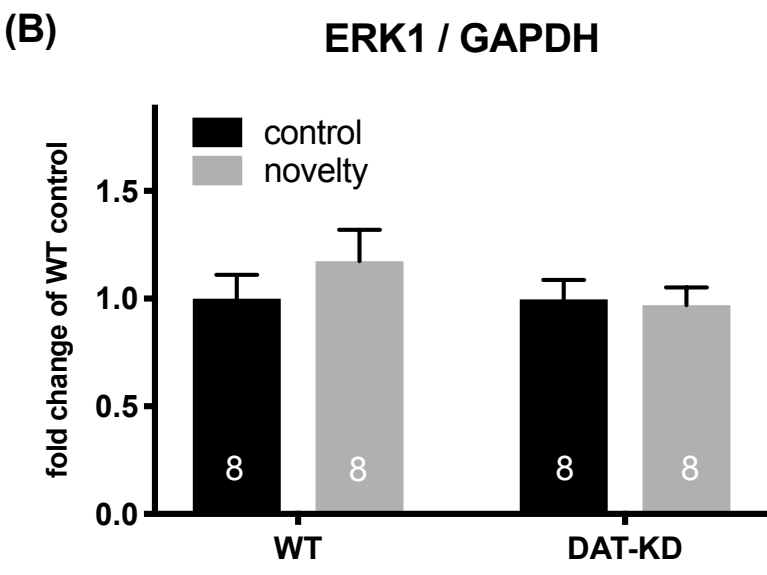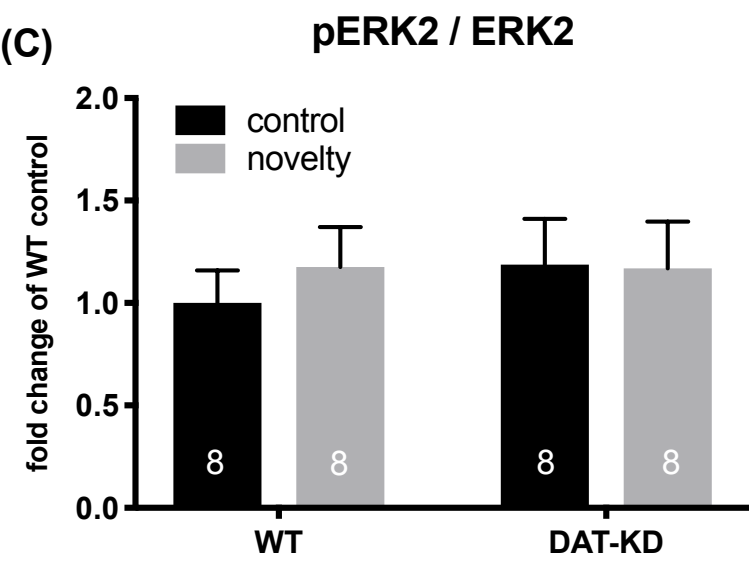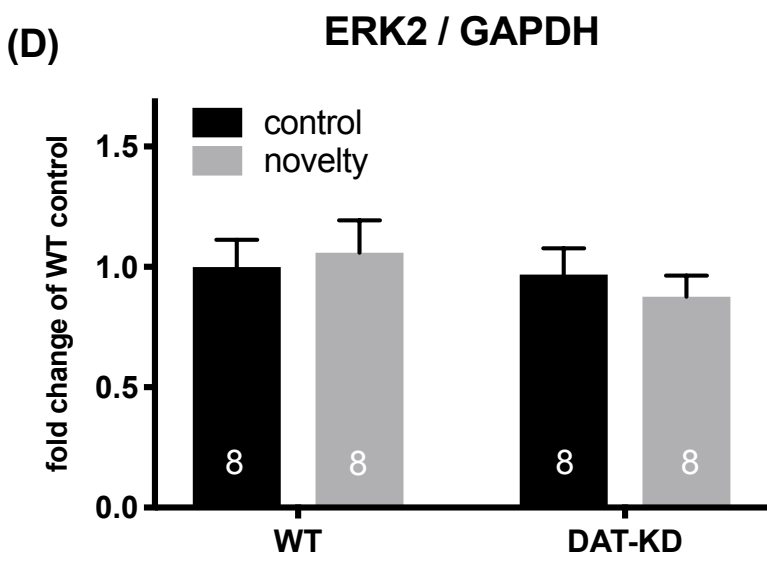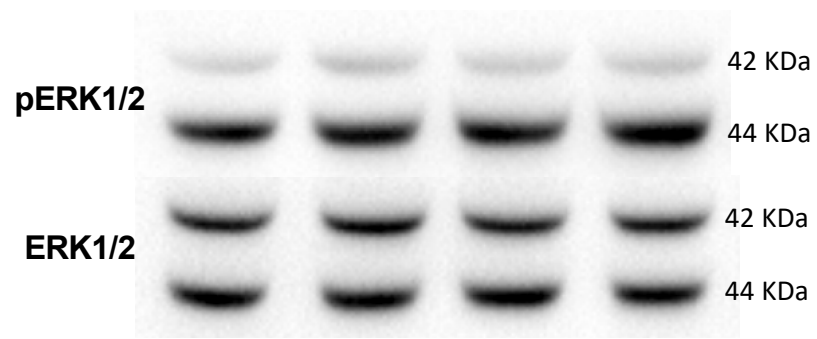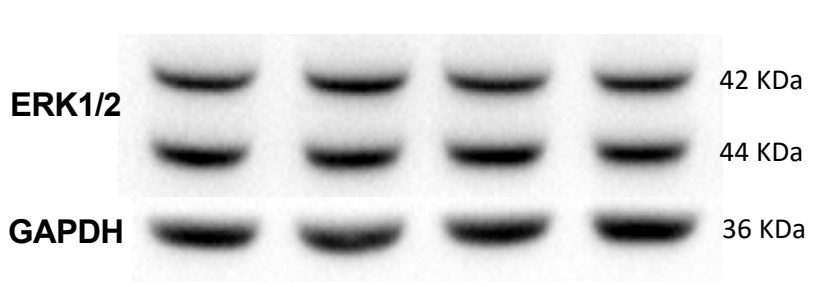

Supplement: Supplementary file 4 — Additional file 4: Figure S4. No effect of novelty exposure on ERK1/2 phosphorylation in the DH. (A) Levels of ERK1 phosphorylation; (B) total amount of ERK1; (C) Levels of ERK2 phosphorylation; (D) total amount of ERK2. Data are shown as mean ± SEM (n = 8 per group). [file 12929_2019_613_MOESM4_ESM.pdf]

**(A) pAkt (s473) / Akt**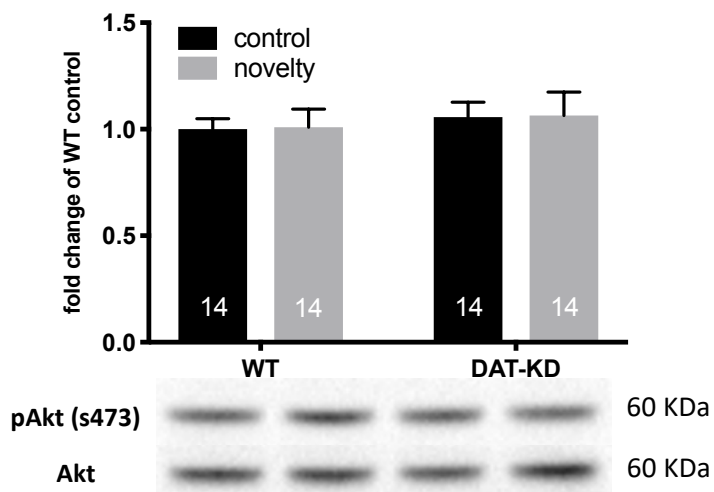**(B) Akt / GAPDH**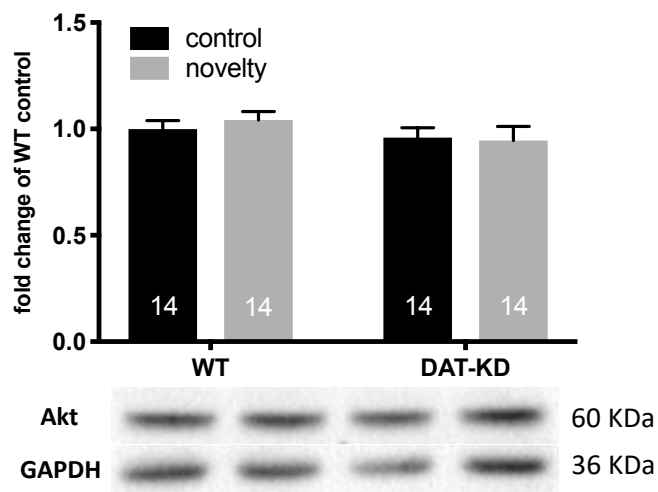**(C) pGSK3 $\alpha$  / GSK3 $\alpha$** 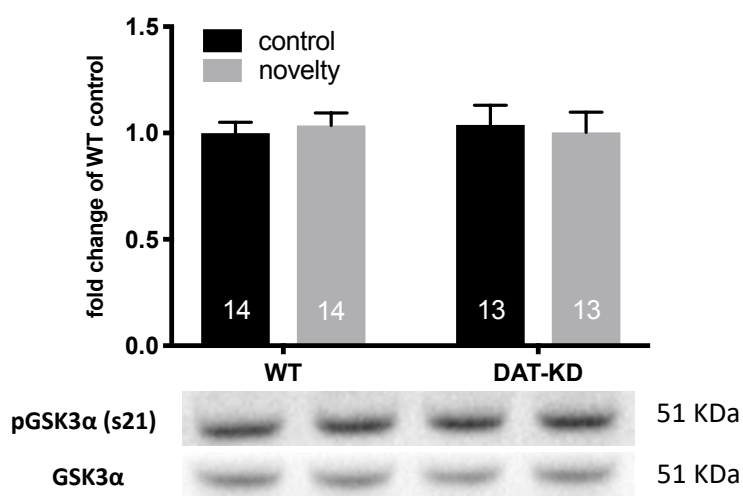**(D) GSK3 $\alpha$  / GAPDH**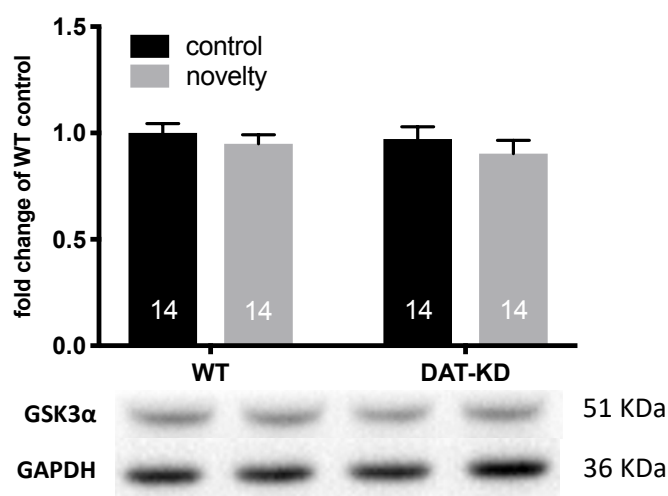**(E) pGSK3 $\beta$  / GSK3 $\beta$** 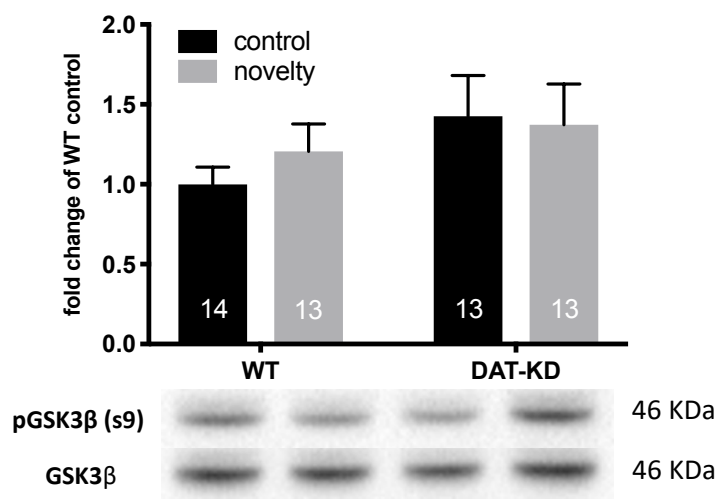**(F) GSK3 $\beta$  / GAPDH**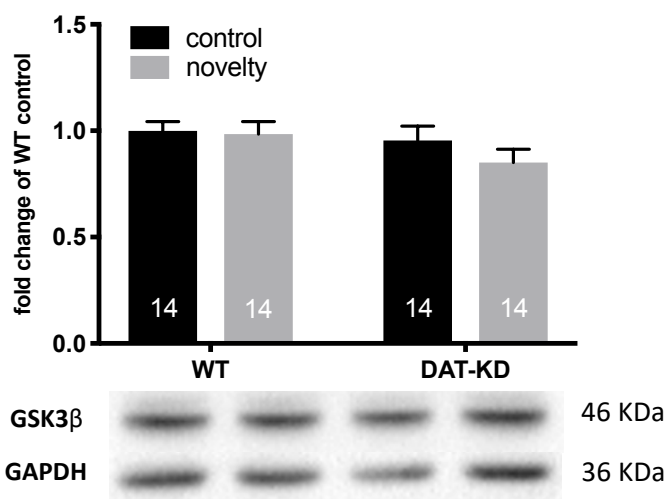

Supplement: Supplementary file 5 — Additional file 5: Figure S5. No effect of novelty exposure on Akt and GSK3 phosphorylation in the VS. (A) Levels of phosphorylation at Akt/serine 473; (B) total amount of Akt; (C) Levels of phosphorylation of GSK3α/serine 21; (D) total amount of GSK3α; (E) Levels of phosphorylation of GSK3β/serine 9; (F) total amount of GSK3β. Data are shown as mean ± SEM (n = 13–14 per group). [file 12929_2019_613_MOESM5_ESM.pdf]

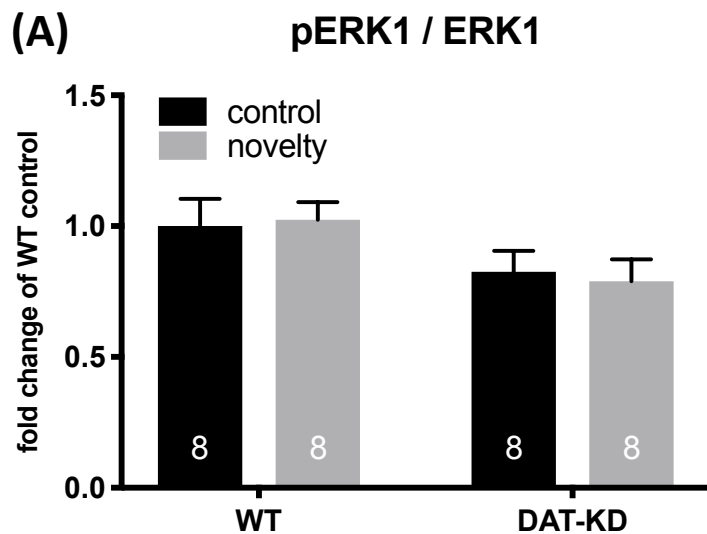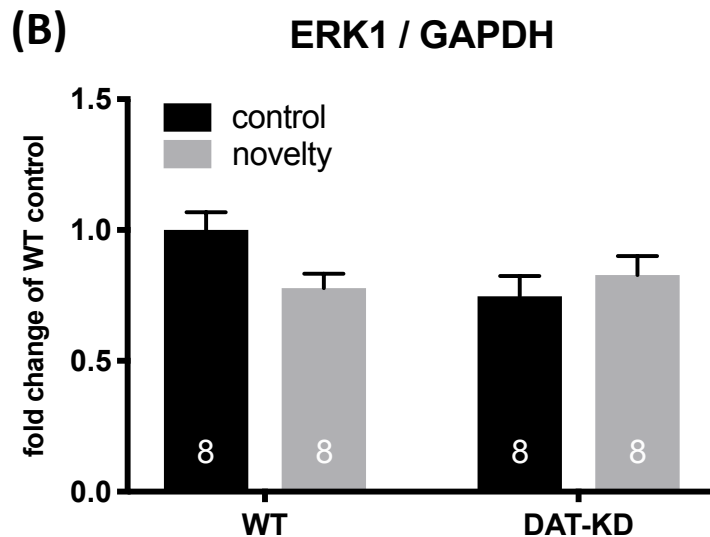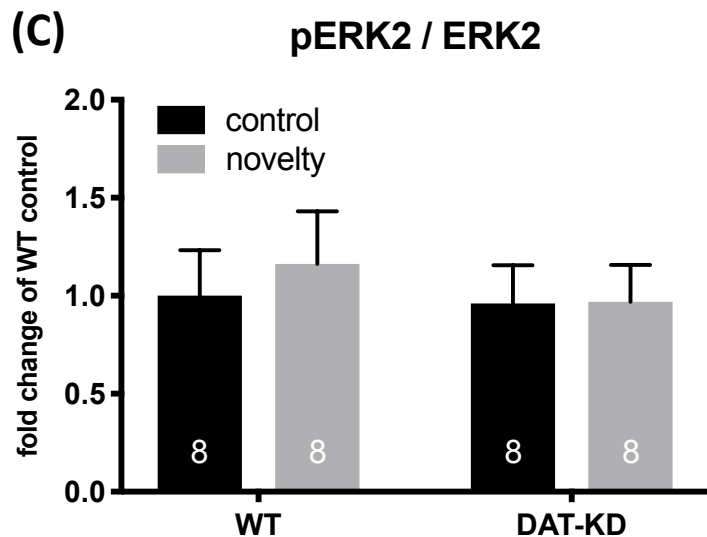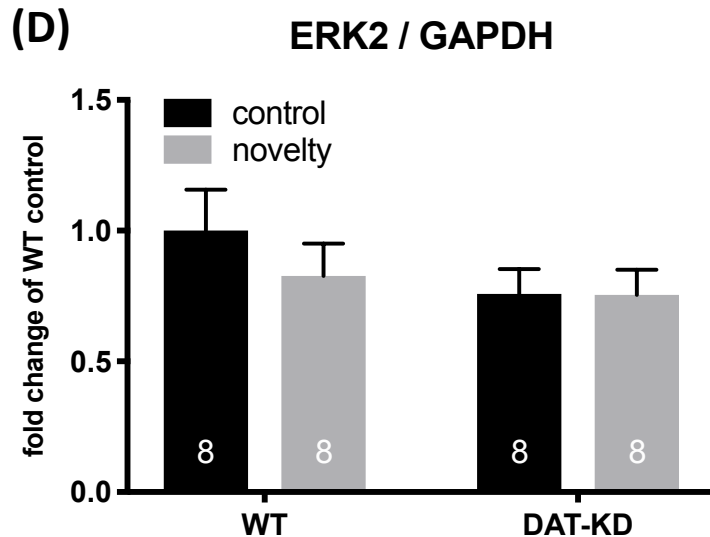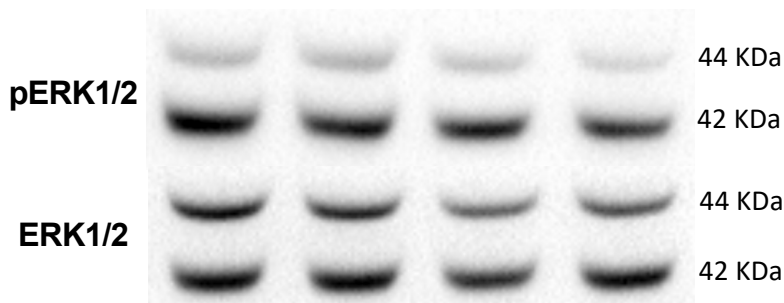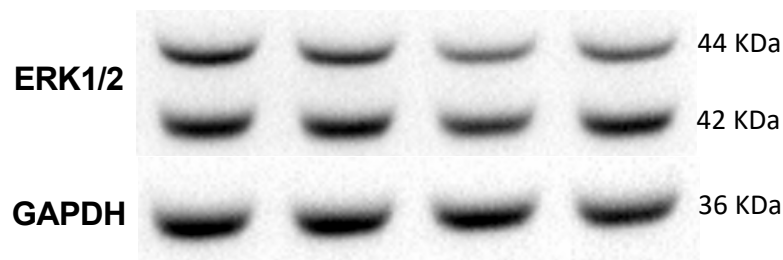

Supplement: Supplementary file 6 — Additional file 6: Figure S6. No effect of novelty exposure on ERK1/2 phosphorylation in the VS. (A) Levels of ERK1 phosphorylation; (B) total amount of ERK1; (C) Levels of ERK2 phosphorylation; (D) total amount of ERK2. Data are shown as mean ± SEM (n = 8 per group). [file 12929_2019_613_MOESM6_ESM.pdf]

**(A)****pAkt(s473) / Akt**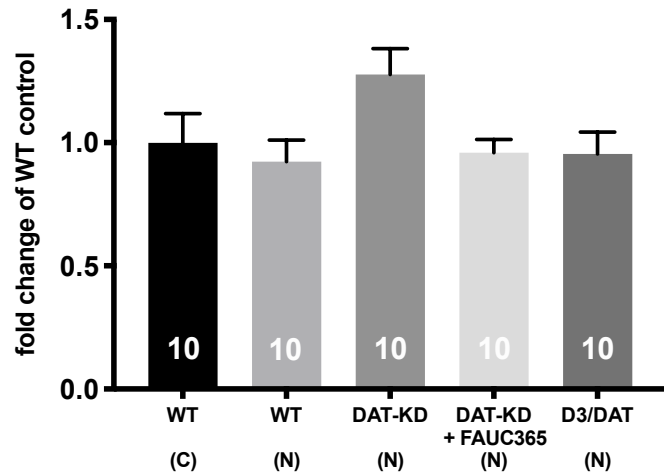**pAkt (s473)****Akt**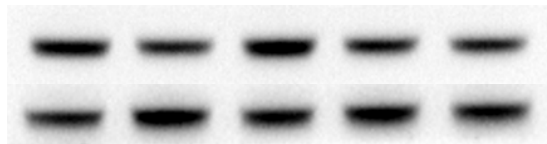

60 KDa

60 KDa

**(B)****Akt / GAPDH**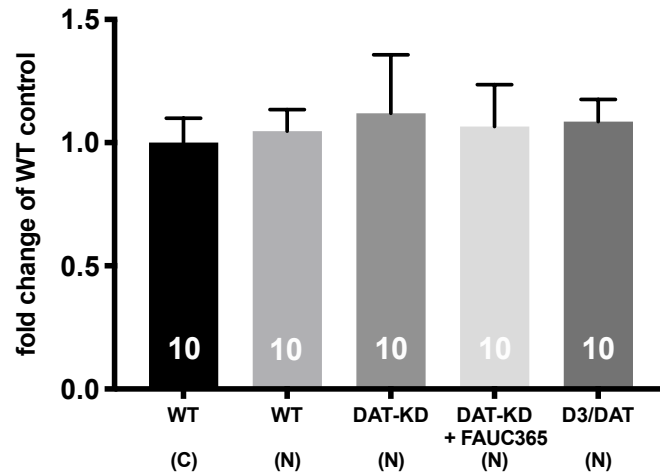**Akt****GAPDH**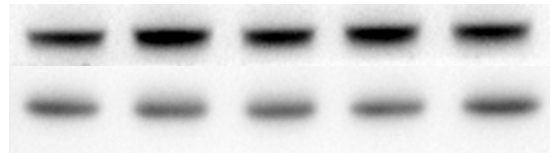

60 KDa

36 KDa

Supplement: Supplementary file 7 — Additional file 7: Figure S7. No effect of D3R inhibition or deletion on Akt phosphorylation in mice mPFC after novelty exposure. (A) Levels of phosphorylation at Akt/s473; (B) total amount of Akt. (n), novelty exposure; (c) control group (n = 10 per group). [file 12929_2019_613_MOESM7_ESM.pdf]

**(A)****C57BL/6**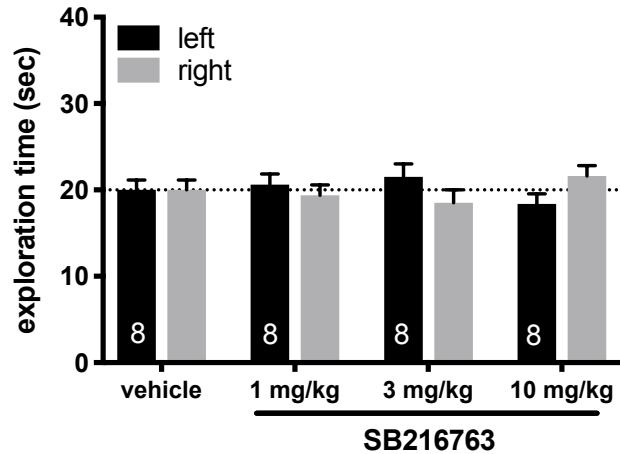**(B)****C57BL/6**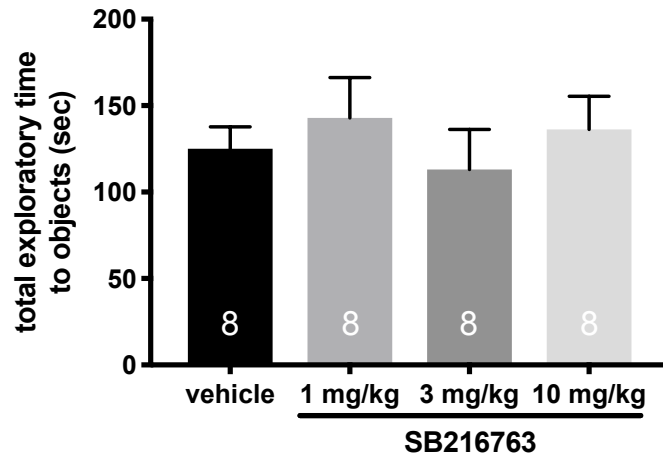**(C)****C57BL/6**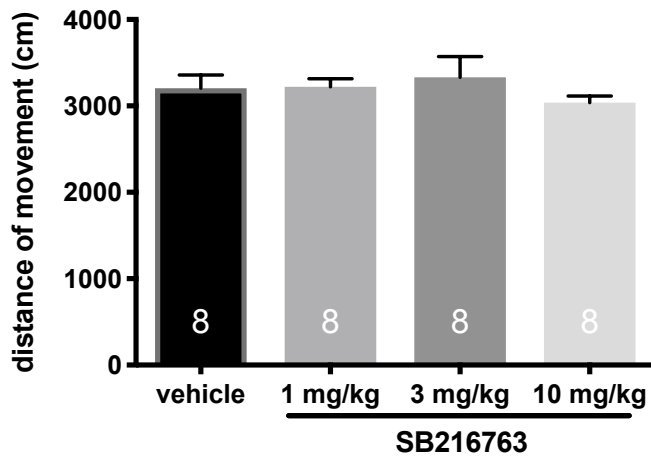

Supplement: Supplementary file 8 — Additional file 8: Figure S8. Effects of SB216763 on the NOR task in mice. (A) Exploration time up to 40 s during the training trial. (B) Total exploration time spent on two identical objects during the NOR training trial. (C) Cumulative horizontal locomotor activity was recorded for a total of 10 min during the NOR training trial. Data are shown as mean ± SEM (n = 8 per group). [file 12929_2019_613_MOESM8_ESM.pdf]

**(A)**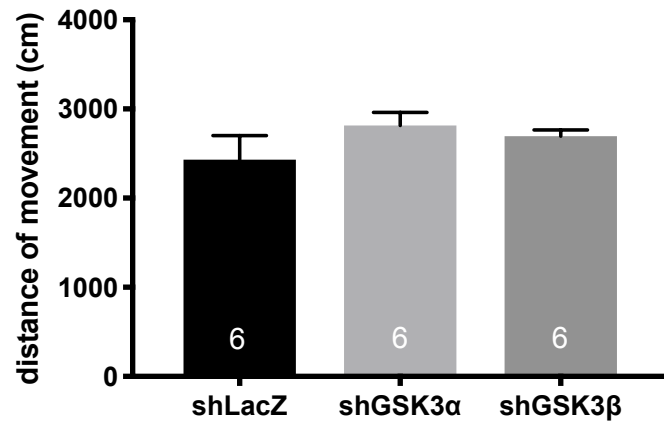**(B)**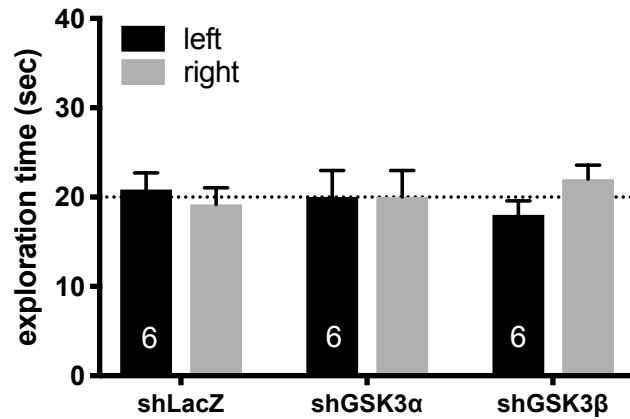**(C)**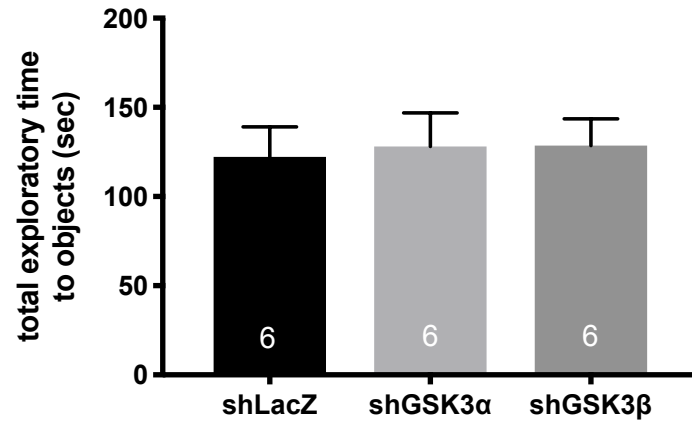

Supplement: Supplementary file 9 — Additional file 9: Figure S9. Effect of knocking down GSK3α or GSK3β on horizontal locomotion and NOR task in mice. (A) Cumulative horizontal locomotor activity was recorded for a total of 10 min during the NOR training trial. (B) Exploration time up to 40 s during the training trial. (C) Total exploration time spent on two identical objects during the NOR training trial. Data are shown as mean ± SEM (n = 6 per group). [file 12929_2019_613_MOESM9_ESM.pdf]

**(A)** pGSK3 $\alpha$  / GSK3 $\alpha$

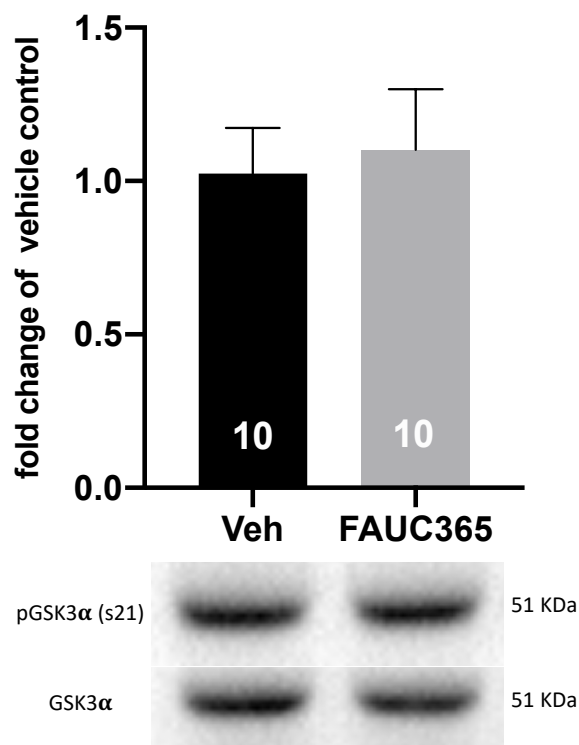

**(B)** pGSK3 $\beta$  / GSK3 $\beta$

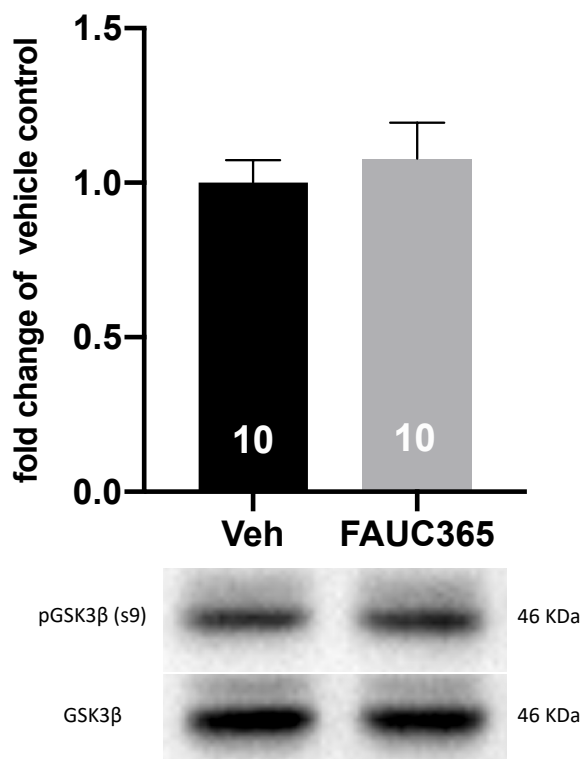

**(C)** GSK3 $\alpha$  / GAPDH

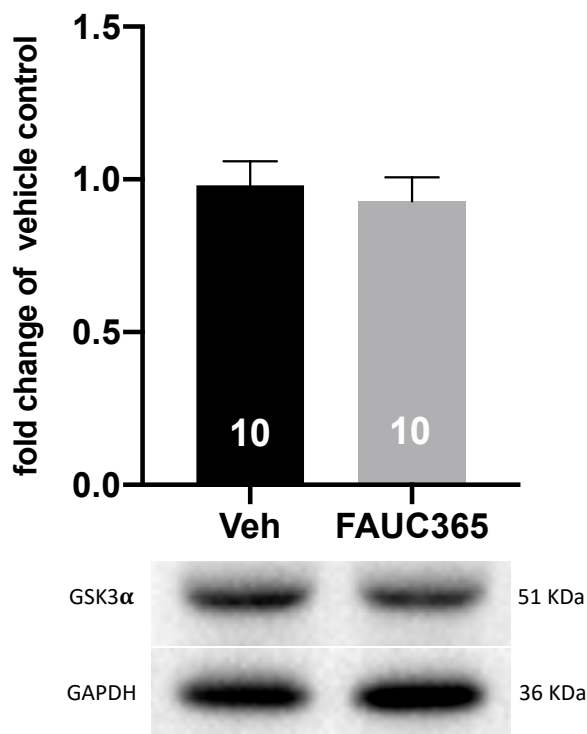

**(D)** GSK3 $\beta$  / GAPDH

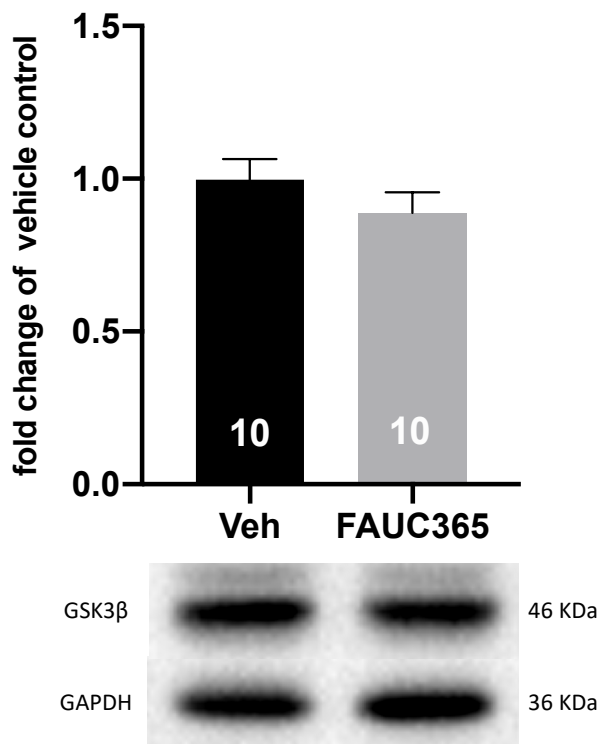

Supplement: Supplementary file 10 — Additional file 10: Figure S10. Phosphorylation and total GSK3 isoforms in C57BL/6 mice that received 3 mg/kg FAUC365 for 10 min. (A) Phosphorylation at GSK3α/serine 21; (B) Levels of phosphorylation at GSK3β/serine 9; (C) total amount of GSK3α and (D) total amount of GSK3β. Data are shown as the mean ± SEM (n = 10 per group). [file 12929_2019_613_MOESM10_ESM.pdf]
